# Effectiveness Analysis of Systematic Combined Sewer Overflow Control Schemes in the Sponge City Pilot Area of Beijing

**DOI:** 10.3390/ijerph16091503

**Published:** 2019-04-28

**Authors:** Yongwei Gong, Ye Chen, Lei Yu, Junqi Li, Xingyao Pan, Zhenyao Shen, Xiang Xu, Qianying Qiu

**Affiliations:** 1 Key Laboratory of Urban Stormwater System and Water Environment, Ministry of Education, Beijing University of Civil Engineering and Architecture, Beijing 100044, China; gongyongwei@163.com (Y.G.); superxyzxyz@126.com (Y.C.); xuxiang1227@163.com (X.X.); 2 Beijing Water Science and Technology Institute, Beijing 100048, China; yl@bwsti.com (L.Y.); pxy@bwsti.com (X.P.); 18811179340@163.com (Q.Q.); 3 State Key Laboratory of Water Environment Simulation, School of Environment, Beijing Normal University, Beijing 100875, China; zyshen@bnu.edu.cn

**Keywords:** combined sewer overflow, hydrological model simulation, control strategy

## Abstract

Combined sewer overflow (CSO) pollution poses a serious threat to the urban water environment and is more severe in old urban areas. This research uses the old urban area in the sponge city pilot area in Tongzhou District, Beijing, as the study area. The United States Environmental Protection Agency (USEPA) storm water management model (SWMM) was used to establish the hydrologic and hydraulic model of this area. The model parameters were calibrated and validated based on the measured rainfall and runoff data. The results show that the Nash-Sutcliffe efficiency coefficient for calibration and validation is more than 0.74. Thirty-two sets of systematic CSO control schemes are formulated, which include the "gray (includes the pipes, pumps, ditches, and detention ponds engineered by people to manage stormwater) strategy" and "gray-green strategies", and the regularity of CSO control for "low impact development (LID) facilities at the source", "intercepting sewer pipes at the midway", and "storage tank at the end", are quantitatively analyzed. The results show that the LID facility has an average annual reduction rate of 22% for the CSO frequency and 35% to 49% for the CSO volume. The retrofitting of intercepting sewer pipes has an average annual reduction rate of 11% for the CSO frequency and 4% to 15% for the CSO volume, and the storage tank has an average annual reduction rate from 3% to 36% for the CSO volume; furthermore, the reduction rate decreases with the increase in the CSO volume reduction rate by LID facilities. When the CSO control target is stricter, the control effect of the "end" segment is more obvious, but the control efficiency is lower. By studying the variability of the storage tank volume under different control targets, it can be concluded that it is reasonable to set the CSO control target because the number of overflow events does not exceed four times per year for the study area.

## 1. Introduction

In the early stages of urban development in various countries, drainage systems were established to collect and transport sewage and stormwater to prevent diseases caused by urban water pollution. Therefore, combined sewer systems (CSSs) were designed to carry sanitary sewage (consisting of domestic, commercial, and industrial wastewater) and stormwater in a single pipe to a treatment facility [1]. To ensure the life and traffic safety of urban residents and avoid the occurrence of urban floods, the design of urban drainage systems adopts the concept of quick drainage. With the establishment and development of drainage systems and the expansion of cities, it is inevitable that the frequency and volume of combined sewer overflow (CSO) and the peak flow in downstream drainage networks will increase dramatically. CSO contains organic substances, pathogens, and toxic substances and has been identified as a significant contributor to water pollution and negative human health impacts [2,3,4]. The problem of CSO pollution has become a great potential danger for urban development [5,6,7]. Therefore, the control of the CSO will become a focus and it will be difficult to control water pollution in the process of urban development both in the present and in the future.

Historically, the control of CSO has proven to be extremely complex. The United States incorporated CSOs into the NPDES (National Pollutant Discharge Elimination System) in 1989 [8], and published the "CSO Guidance for Long-Term Control Plan" and "Guidance for Nine Minimum Controls" [9,10]. Studies by Marengo et al. showed that most of the methods to control CSOs include increasing the conveyance capacity of drainage systems and the storage capacity of storage facilities [11]. It has been shown that gray (includes the pipes, pumps, ditches, and detention ponds engineered by people to manage stormwater) strategies can be effective, and storage tanks represent the most traditional type of CSO control facility [12,13]. The research results of Fu et al. supported that the rational optimization of the storage tank can alleviate the current situation of urban water pollution [4]. Phillips et al. showed that reducing rainwater runoff, increasing pipeline conveyance capacity, and storing peak flow during rainfall are the main factors to consider in CSO control [14]. In addition, in the gray strategy, the construction of intercepting sewer pipes is also an effective method to control CSO [15]. In a case of CSO control in Port Angeles [16], the conclusion indicated that the intercepting sewer pipe had the function of storing and discharging part of the stormwater runoff. However, there are some problems in the construction of such gray facilities, such as difficulties in site selections, high costs, and limited land conditions [12].

In the early 20th century, urban stormwater management began to advocate green stormwater infrastructure (GSI), or so-called low impact development (LID) measures [17]. Previous studies show that LID facilities can effectively reduce CSO [14,18,19,20]. At the same time, GSI or LID facilities have become alternatives to gray facilities in terms of controlling CSO [21]. Since LID can effectively restore original hydrological conditions and remove pollutants from stormwater runoff [22,23,24], it is superior to the traditional gray facilities from the perspectives of the economy, environment, and society [25]. The gray-green CSO pollution control strategy is to reduce the runoff volume entering the CSSs by building LID facilities at the source [26]. Franco Montalto et al. found that phasing LID systems into the watershed was a more cost-effective strategy for reducing CSO than building large-sized CSO tanks [12]. In his cases, the lowest cost path for reducing CSO relied exclusively on LID technologies and involved the construction of tanks only after all LID opportunities had been exhausted if additional reductions in CSO were sought.

Many studies have compared the cost-effectiveness of the gray and gray-green combination control methods in different categories and have concluded that the gray-green combined alternative is more cost-effective than the gray-only option [7,27]. Sebti et al. proved that the application of best management practice (BMP) facilities can effectively improve the CSO control effect in the process of model optimization studies on the BMP and combined networks [28].

In recent research on CSO control strategies, more attention has been paid to the CSO control effect when LID facilities, intercepting sewer pipes, or storage tanks are implemented alone. There is a lack of research on the “source-midway-end” systematic CSO control scheme in a CSS area. “Source” refers to building LID facilities to reduce stormwater runoff, "midway" refers to setting up a reasonable interception rate and a reasonable size of intercepting sewer pipes to improve the drainage capacity of the network, and "end" refers to setting up storage tanks at the outlet of the CSS to reduce or completely control the CSO.

This paper formulated systematic CSO control schemes according to different “source” control targets, intercepting sewer pipe collocation forms, and volumes of storage tanks. The storm water management model (SWMM) of the study area is built up, and the regularity of the CSO reduction rate in each segment of the systematic scheme is studied.

## 2. Materials and Methods

### 2.1. Study Area Description

The old urban area and the newly built area are included in the sponge city pilot area in Tongzhou District, Beijing. The size of the old urban area is 741 ha. According to the topological structure of the pipe network of the research area, it is divided into six drainage zones, S1–S6, of which S3 is a typical combined sewer system area. CSO pollution is more prominent in the S3. This paper selects a typical CSS area within the pilot area and formulates a systematic control strategy for CSO pollution using SWMM.

**Figure 1:**
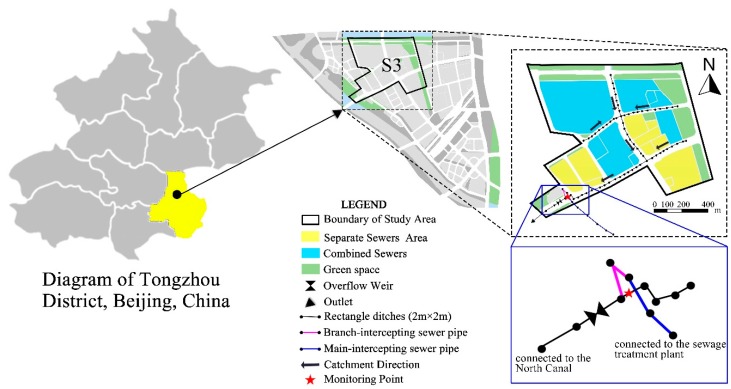
General situations of the study area.

The old urban area is divided into six drainage zones based on the factors of topography, elevation, ground slope and pipe network layout, among which the study area is in the S3 zone. The study area covers an area of 84.09 ha, of which the impervious area is 51.06 ha and mainly includes buildings and roads. The location of the study area is shown in Figure 1. The climate of the study area is a temperate zone continental monsoon climate. The average annual temperature is 14.6℃, the average annual precipitation is 535.9 mm, and the average annual evaporation is 1308 mm. The precipitation in the flood season (i.e., June–August) accounts for more than 80% of the annual precipitation. The soil in the study area mainly includes silty clay, fine sand, medium sand, and coarse sand. The comprehensive vertical permeability coefficient ranges between 6.13 × 10^−4^ cm/s and 1.41 × 10^−3^ cm/s, and the comprehensive horizontal permeability coefficient ranges between 4.00 × 10^−3^ cm/s and 1.57 × 10^−2^ cm/s.

The main underlying surface of the study area includes residential land, public facility land, green space, and roads. The terrain in the area is flat. The drainage forms of the study area are shown in Figure 1.

The CSO pollution in the study area is the main problem threatening the water environment of the river. As shown in Figure 1, there is only a municipal stormwater pipe in the study area. When the pipeline network of the combined sewer area is connected to the municipal stormwater pipe, the stormwater is mixed to form the combined sewage material. The sewage overflows from the drainage outlet during wet weather, and the volume of the sewage is very large; thus, these overflows influence the river water quality. The municipal stormwater pipes underneath the ground are rectangle ditches with a cross-section of 2 m × 2 m, and the drainage outlet of the municipal pipe network is connected to the North Canal. To control CSO, intercepting sewer pipes and overflow weirs have been constructed at the outlet. The branch-intercepting sewer pipe with a diameter of 500 mm is connected at 0.3 m from the bottom of the drainage channel, and the main-intercepting sewer pipe with a diameter of 800 mm is connected downstream. The main-intercepting sewer pipe is connected to the intercepting sewer pipe downstream with a diameter of 1100 mm, and it eventually discharges into the sewage treatment plant. Meanwhile, an overflow weir with a height of 0.8 m was installed near the outlet. In wet weather, the capacity of the intercepting sewer pipes is not enough to transfer excessive combined sewage. When the water level in the pipeline reaches 0.8 m, the combined sewage is discharged into the river through the overflow weir, which results in CSO pollution.

### 2.2. Data Collection

The rainfall data are monitored by the Meteorological Bureau of Tongzhou District, Beijing. These data are recorded every five minutes. The simulation selects the complete rainfall data from 1 January 2013 to 30 November 2017. The statistical results of precipitation in Beijing from 1952 to 2017 show that wet years and dry years alternate, and the period of 2013–2017 was considered a wet period. This period had a higher CSO control standard to formulate the CSO systematic control schemes by using rainfall in wet years as input conditions. Statistical analysis of the rainfall data from 2013 to 2017 was carried out. The results showed that there were 48, 41, 46, 46, and 30 rainfall events each year, respectively. The amount of rainfall in the maximum rainfall event each year was 64.4, 126.1, 130.9, 224.2, and 109.4 mm, respectively. The largest rainfall events occurred from June to September each year. The average duration of the rainfall events each year was 14, 11, 19, 11, and 16 h, respectively.

The water level of the municipal stormwater main pipe at the outlet of the research area was monitored, and the monitoring point was located in the rectangle ditches (with a cross-section of 2 m × 2 m) near the outlet. The specific location of the monitoring point is shown in Figure 1.

The four rainfall events (Table 1) with complete data were selected and used to calibrate and verify the main hydrological parameters of the model. Among them, the rainfall events on 26 June 2018 and 7 July 2018 were used for model calibration, and the rainfall events on 11 July 2018 and 16 July 2018 were used for model validation.

### 2.3. Model Set Up and Evaluation Criteria

This study employs long-term continuous hydrologic and hydraulic simulations using the SWMM. The SWMM of the study area was established based on the land use status, drainage network layout, and ground elevation. The study area can be generalized into 95 sub-catchment areas, with 51 rainwater pipe sections, 104 manholes, and 2 drainage outlets.

The Nash-Sutcliffe Efficiency coefficient (E_NS_) is used to determine the accuracy of the simulation results of the hydrological model, and this coefficient has a value between -∞ and 1. An E_NS_ value close to 1 indicates the simulation results have high credibility [8].

## 3. Design of CSO Pollution Control Schemes

### 3.1. CSO Pollution Control Target

The study area is the only national sponge city pilot area in Beijing, and it is located in Tongzhou District. When the co-development of Beijing-Tianjin-Hebei became a national strategy, Tongzhou District of Beijing was selected for the site of the Beijing City Sub-Center, whose purpose was to alleviate non-capital features. In view of Tongzhou’s important strategic position in terms of development, advanced design concepts and high-quality construction standards should be applied in the construction of national sponge city pilot areas. Therefore, under the situation of serious CSO pollution in the study area, it is necessary to formulate international advanced levels of CSO control targets and strategies.

Currently, there are no clear laws or regulations that control CSO pollution in China. The target for controlling CSO is defined as achieving an annual overflow frequency less than 15 times in certain pilot projects of sponge cities in southern China; however, the rainfall and CSO frequency in this study area are obviously different from those in southern cities. According to the information provided by the staff of Tongzhou Water Administration, the frequency of CSO in the study area is approximately 10 times per year, which is much lower than that of the southern cities. At the same time, referring to the CSO control targets and management experience of the United States, the average CSO frequency control targets of Georgia, New York, Missouri, Indiana, and Nebraska are no more than four times per year. Therefore, combined with the current situation of CSO pollution in the study area, the annual average frequency of CSO should not exceed four times in the study area. The rationality of this control target is discussed in Section 4.4.2.

### 3.2. Design of Control Schemes

The CSO control strategies are chosen according to the construction conditions of different cities. Combined with the experience of controlling CSO in foreign countries, this area needs to systematically design and construct schemes that combine source control facilities and midway pipeline networks; additionally, schemes should be formulated to combine LID facilities at the source, intercepting sewer pipes at the midway, and the storage tanks at the end. Each segment of the initial CSO control scheme is introduced below.

For the source control strategy, there are three methods used to determine the storage capacity of LID facilities. ① According to the Beijing local standard "Code for design of stormwater management and harvest engineering (DB11/685—2013)", it has the following specific requirements "for each 1000 m^2^ of impervious area, a stormwater storage facility with a capacity of 30 m^3^ should be built at least; for construction projects that affect the green space requirements, more than 50% of green space should be lower than the surrounding ground; furthermore, the rate of permeable pavement in public parking lots, sidewalks, pedestrian streets, bicycle lanes, leisure squares and outdoor courtyards should be no less than 70%". ② According to the 75% volume capture ratio of annual rainfall target, the storage volume at the source can be determined. ③ The storage volume at the source is determined according to the actual survey results. The determination of the above three methods has practical significance. Method ① is to determine the storage capacity of LID facilities according to the "Code for design of stormwater management and harvest engineering (DB11/685—2013)" during the construction of the new district. Method ② is the fundamental objective of the sponge city pilot area in Tongzhou District. Method ③ makes full use of the remaining space of the old city to build LID facilities. The operability of Method ③ lies in the fact that construction space will restrict the construction of LID facilities in practical projects. To ensure that the area can achieve the overall goal of stormwater control, developers can offset their excess runoff by purchasing approved credits generated elsewhere [29]. Combining the above stormwater management concepts, in terms of achieving the overall target for the pilot area, the scheme is designed based on local conditions. The storage capacity of the three strategies gradually decreases from strategy ① to strategy ③, which meets the needs of different background conditions in the old urban area and the new urban area. The strategies are shown in Table 2.

For the mid-way control strategy, the retrofitting of the intercepting sewer pipes can maximize the amount of combined sewage that is discharged into a sewage treatment plant by improving the interception capacity. Therefore, this project will expand the existing intercepting sewer pipes under Dongbinhe Road and design several combination schemes of different diameters for the main- and branch-intercepting sewer pipes. The design principle for retrofitting the intercepting sewer pipes states that the diameter of the branch-intercepting sewer pipes should not exceed the diameter of the main-intercepting sewer pipes, and the largest diameter of the main-intercepting sewer pipe is 1000 mm. It is noteworthy that the design of the intercepting sewer pipes in this paper considers only the effect of the change in the diameter of the intercepting sewer pipes on CSO and does not involve the design flow and the treatment capacity of the sewage treatment plants. The retrofitting scheme of the intercepting sewer pipes is shown in Table 3.

Thirty-two schemes were formulated by combining four schemes of LID construction (one without LID facilities) with eight schemes of retrofitting the pipeline network. According to the source schemes, the 32 schemes were divided into four types, including type I (schemes 1 to 8), type II (schemes 9 to 16), type III (schemes 17 to 24), and type IV (schemes 25 to 32), as listed in Table 4.

For the "end" control strategy, if the SWMM simulation results show that the CSO control target can be achieved under the combined scheme of LID facility construction and retrofitting of intercepting sewer pipes, there is no need to build storage tanks; otherwise, it is necessary to construct a storage tank to control the CSO. The method for determining the volume of a storage tank is as follows: the SWMM was used to simulate the frequency and volume of CSO based on the rainfall data from 2013 to 2017 after the application of the 32 control schemes. When the simulation results showed that the average annual overflow frequency was more than four times per year, the CSO volume of the different schemes in different years was arranged in descending order, and the construction volume of the CSO storage tank was the average of the fifth CSO volume in each year. The volume of storage tanks for each scheme will be calculated in Section 4.3.

## 4. Results and Discussion

### 4.1. Model Calibration and Validation

**Figure 2:**
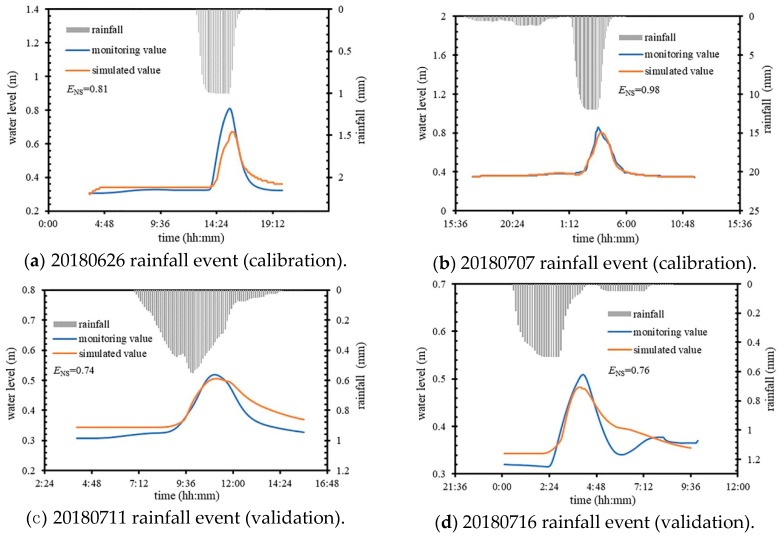
Calibration and validation of model parameters in the study area (the E_NS_ is used to determine the accuracy of the simulation results of the hydrological model).

The model was calibrated and validated using the monitored drainage trench water level data. The E_NS_ between the monitored water level values and the model simulation values after stabilization were calculated, with values of 0.81, 0.98, 0.74, and 0.76, respectively. The calibration and validation performance of the model parameters are shown in Figure 2.

The rainfall events used for model calibration and validation ranged between 11 mm and 30 mm, and the rainfall duration was between 3.5 and 23.8 h, which covers both moderate and heavy rainfall events. Under these four rainfall events, the maximum water level of the pipeline ranged 0.51–0.85 m, and the simulation results of the model in the study area were well-fitted to the monitored values. Figure 2a,c,d shows that there are some differences between the monitored and simulated water levels at the beginning and end stages of rainfall events. The difference in the beginning stage of rainfall events was mainly due to the short warm up time of the model, and the difference in the end stage of rainfall events was mainly due to the large fluctuation of the actual water level and the instability of monitoring data. In general, the calibration and validation results of the model were satisfactory, and the model can be used for subsequent research and analysis.

### 4.2. Simulation Results of Current CSO Conditions

The frequency and volume of CSO under the current situation was simulated using the observed rainfall data from 2013 to 2017. The rainfall data used 24 h with no or negligible rainfall to separate the time series into individual events. In the process of actual rainfall and simulation, CSO may occur many times during a single rainfall event. Therefore, this study defines multiple overflows in a single rainfall event as one CSO event. The statistical results of the model simulation showed that the number of annual CSO events in the years 2013–2017 was 9, 7, 7, 12, and 10 times, respectively, and the average annual CSO volume in each of the five years was 27.8 × 10^3^ m^3^, 71.1 × 10^3^ m^3^, 42.7 × 10^3^ m^3^, 109.9 × 10^3^ m^3^, and 102.5 × 10^3^ m^3^, respectively. The simulation results showed that the average annual frequency of CSO over all five years was 9. The annual overflow was approximately 70.8 × 10^3^ m^3^. There were great differences in the CSO in different years, among which the largest was 109.9 × 10^3^ m^3^ in 2016 and the lowest was 27.8 × 10^3^ m^3^ in 2013. The difference in annual overflows was mainly dependent on the precipitation and the frequency of storm events.

The general trend of the annual overflow in these years was that there was a higher total annual overflow when there was a greater number of overflow events; however, there were differences in individual years. For example, the frequency of CSO in 2013 was more than that in 2014, but the total volume of the overflows was 43.0 × 10^3^ m^3^ less in 2013 than that in 2014. According to the statistical results, the total volume of CSO from 9 CSO events in 2013 was 27.8 × 10^3^ m^3^, and the maximum volume of overflow from a single CSO event was 8.0 × 10^3^ m^3^. The maximum volume of overflow from a single overflow event in 2014 was 30.9 × 10^3^ m^3^, which was higher than the total volume of CSO from the whole year of 2013 and far exceeded the maximum volume of overflow from a single CSO event in 2013. The analysis of rainfall data showed that there was a higher number of rainfall events in 2013, but the average volume of each rainfall event was lower; in contrast, the opposite trend was observed in 2014, which explains the results described above. Compared with the simulation results in 2014 and 2015, the annual overflow discrepancy was nearly 28.0 × 10^3^ m^3^ below the same overflow frequency, which also illustrates this phenomenon. These two elements (CSO frequency and volume) will be discussed in the process of CSO control scheme optimization.

### 4.3. Simulation Results of Control Schemes without Storage Tanks

**Figure 3:**
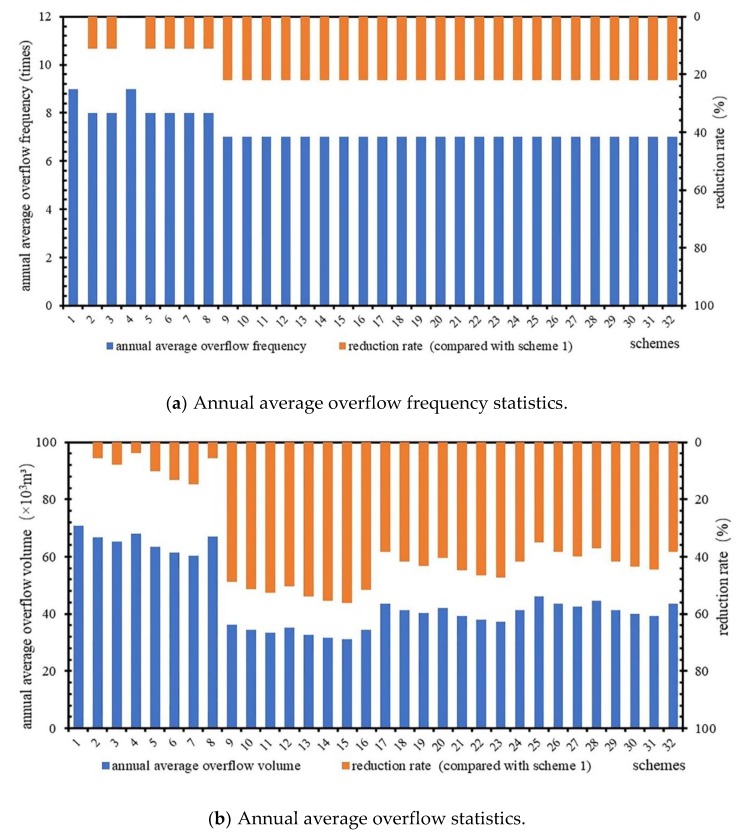
Simulation results of scheme without extra storage tanks (source + midway).

Using the rainfall data from 2013 to 2017, 32 schemes were simulated without storage tanks to obtain the frequency and volume of CSO, and the results are shown in Figure 3.

Figure 3 shows that the reduction rate of the annual average overflow frequency of scheme 4 in type I is 0 and that of the other schemes is 11%. In type II, III, and IV, the reduction rate of the overflow frequency of each scheme was 22%. It was difficult to identify the differences in the CSO control effects among scheme by comparing only the reduction rate of overflow frequency; thus, it was necessary to compare the average annual overflow among the different schemes. Figure 3 shows an average CSO reduction rate of 37% for all schemes, of which scheme 4 has a maximum overflow of 68.1 × 10^3^ m^3^ and a reduction rate of only 4%. The annual overflow of scheme 15 was the smallest, at 31.1 × 10^3^ m^3^, and the reduction rate was 56%. In type I, the range of annual overflow of each scheme was between 60.4 × 10^3^ and 70.8 × 10^3^ m^3^, the average value was 65.4 × 10^3^ m^3^, and scheme 7 had the highest reduction rate of 15%. In type II, the range of annual overflow of each scheme was between 31.1 × 10^3^ and 36.3 × 10^3^ m^3^, the average value was 33.6 × 10^3^ m^3^, and scheme 15 had the highest reduction rate. In type III, the range of annual overflow of each scheme was between 37.3 × 10^3^ and 43.7 × 10^3^ m^3^, the average value was 40.4 × 10^3^ m^3^, and scheme 23 had the highest reduction rate of 47%. In type IV, the range of annual overflow of each scheme was between 39.3 × 10^3^ and 46.1 × 10^3^ m^3^, the average value was 42.6 × 10^3^ m^3^, and scheme 31 had the highest reduction rate of 44%.

Because none of the "source + midway" schemes could reach the control target of fewer than 4 overflow events per year, it was necessary to add a storage tank in each scheme to achieve the CSO control target. The volume of the storage tank was determined as described in Section 3.2; thus, the final scheme was formulated by adding the storage tank to the initial scheme (Table 5). Comparing type I to type IV, if type I (without LID facilities) aims to achieve the CSO control target, it requires storage tanks with the largest volumes, while type II (with the largest storage capacity of LID facilities) requires storage tanks with the smallest volumes. The volumes of storage tanks for schemes 1, 9, 17, and 25 were the largest of each type, respectively. The volumes of storage tanks of schemes 7, 15, 23, and 31 were the smallest of each type, respectively.

**Figure 4:**
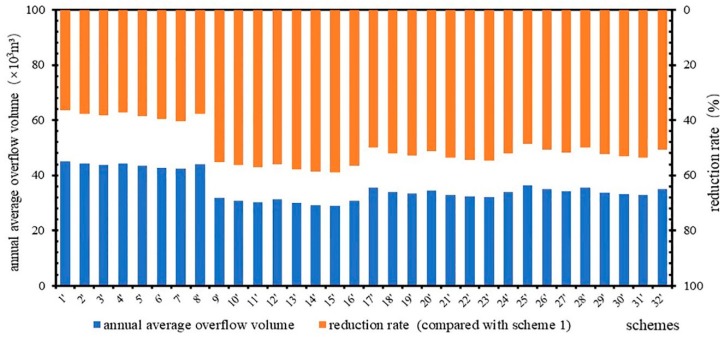
Annual average overflow statistics.

The final 32 schemes can be sorted as “gray strategy (type I)” and “gray-green strategies (types II, III, IV)”. Thirty-two sets of final control schemes with storage tanks were simulated. The average annual CSO of these schemes is shown in Figure 4.

Figure 4 shows that the amount of overflow is different. After increasing the volume of the storage tank, the range of annual overflow among the different schemes narrowed. The coefficient of variation (C_V_) of annual overflow volume of each scheme in Figure 3 is 0.27 and that of Figure 4 is 0.14. The reason for the different volumes of storage tanks in the different schemes is that the purpose of using storage tanks is to achieve the control target. On the premise that all the schemes could meet the CSO control target, the average reduction rate of CSO in all schemes is 50%, which is 13% higher than that without the use of storage tanks. The annual overflow of scheme 15 was the smallest, at 29.0 × 10^3^ m^3^, with a reduction rate of 59%, which was 3% higher than that without a storage tank. In type I, the range of annual overflow of each scheme was 42.3 × 10^3^–45.0 × 10^3^ m^3^, the average value was 43.7 × 10^3^ m^3^, and scheme 7 had the highest reduction rate of 40%, which was 25% higher than that without a storage tank. In type II, the range of annual overflow of each scheme was 29.0 × 10^3^–31.7 × 10^3^ m^3^, the average value was 30.4 × 10^3^ m^3^, and scheme 15 had the highest reduction rate. In type III, the range of annual overflow of each scheme was 32.1 × 10^3^–35.5 × 10^3^ m^3^, the average value was 33.6 × 10^3^ m^3^, and scheme 23 had the highest reduction rate of 55%, which was 8% higher than that without a storage tank. In type IV, the range of annual overflow of each scheme was 32.9 × 10^3^–36.3 × 10^3^ m^3^, the average value was 34.5 × 10^3^ m^3^, and scheme 31 had the highest reduction rate of 54%, which was 10% higher than that without a storage tank.

Compared with the schemes before and after the addition of storage tanks, the control effect of the storage tanks reduced the CSO volume by 3–36%. The main reason for this difference was that all schemes needed to achieve the same control targets. The average CSO reduction rates of type I to type IV were 31%, 5%, 10%, and 12%, respectively. There was no LID facility implemented in type I, and the storage tank required a large volume to achieve the control target. Therefore, the storage tank plays a major role in the control of CSO in such systematic control schemes. Type II to type IV implemented LID facilities; thus, the volumes of storage tanks required for these systems were small. In type II to type IV, the CSO reduction rate by the LID facilities gradually decreased, which caused the CSO reduction rate due to the use of storage tanks to gradually increase.

### 4.4. Regularity Analysis

#### 4.4.1. Effect of LID and Pipe Retrofitting

To separately study the CSO control effect of LID facility construction and retrofitting intercepting sewer pipes and analyze the differences between them, the regularity of the CSO frequency and volume was analyzed only when LID facility construction or intercepting sewer pipe retrofitting was implemented in the area. The schemes analyzed in this section do not include the construction of storage tanks.

1. CSO control effect by LID facilities

Using scheme 1 as the background reference, the differences in the CSO frequencies and volumes of schemes 9, 17, and 25 were compared to study the control effect of different LID facility layouts. These three sets of schemes did not implement retrofitting of intercepting sewer pipes.

From Figure 3, we can see that the frequency reduction rates of the three schemes are all 22%. The annual overflow reduction rates of scheme 9, scheme 17, and scheme 25 were 49%, 38%, and 35%, respectively. The average reduction rate of annual overflow was 41%. The above conclusions showed that LID facilities had great control effects on the volume of CSO. Montalto et al. proved that the implementation of LID facilities, such as green roofs, porous pavement, and curbside channels/treatment wetlands, could reduce CSO by 26%, 11%, and 10%, respectively [12]. De Sousa et al. used the SWMM to simulate the CSO reduction rate after implementing a combined scheme of LID facilities, such as permeable pavement, biological detention facilities, and rainwater gardens, in their study area [30]. The results showed that LID facilities could reduce the number of CSO events from 12 to 7 and reduce the volume of CSO by 352.0 × 10^3^ m^3^ per year. Tao et al. quantitatively assessed the performance of GSI in controlling urban CSO and the maximum volume reduction rate was approximately 44.13% over a one-year period with the assumption that 100% of generated stormwater runoff was transported from the impervious surface area to the GSI systems [31].

From the above analysis, it can be seen that the CSO control effect of the three green schemes gradually decreased from type II to type IV because the storage capacity at the source decreased from type II to type IV. Type II distributed the storage space at the source according to the "Code for design of stormwater management and harvest engineering (DB11/685—2013)", which maximizes the utilization of the source space and contains a storage capacity up to 56.0 × 10^3^ m^3^; however, this design is difficult to implement in old urban areas. Type III distributed the storage space according to the "volume capture ratio of annual rainfall" in the old urban area, which contained a storage volume of approximately 25.0 × 10^3^ m^3^, and although the control effect was not as good as that of type II, it was more suitable for the old urban area in terms of controlling CSO. The storage capacity of type IV was the smallest of the three sets, at only 19.0 × 10^3^ m^3^. To reduce the CSO volume by 1% per year, the storage capacities required by these three source types were 1.1 × 10^3^ m^3^, 0.7 × 10^3^ m^3^, and 0.5 × 10^3^ m^3^, respectively. Type IV distributed the storage space according to the principle of adjusting measures to local conditions; thus, this type was the easiest to achieve, and its volume control effect was only 3% different from that of type III. Tao et al. showed that the control effect of GSI was related to the amount of runoff transferred to the GSI units [31]. In practical projects, LID facilities cannot control all runoff generated from impervious surfaces. Therefore, it is unreasonable and wasteful to infinitely increase the storage capacity of LID facilities.

Based on the above conclusions, in a large-scale area such as the China Sponge City Construction Pilot Project, it is necessary to rationally select and establish LID facilities at the source based on the local water environment, water ecological construction objectives, and restrictions. Although the LID facility has a positive effect on CSO reduction, CSO may still occur during or after some small rainfall events. Therefore, when exploring a systematic CSO control scheme, the CSO reduction regularity in the "midway" and "end" points are still of great significance.

2. CSO control effect by retrofitting intercepting sewer pipes

From Figure 3 (CSO control schemes without storage tanks), we can see that the frequency reduction rate of schemes 2 to 8 is 0–11%, the average reduction rate is 9%, the annual overflow reduction rate is 4–15%, and the average reduction rate is 9%. In this regard, the CSO control efficiency of enlarging the intercepting sewer pipes was lower than that of constructing LID facilities. The United States has implemented several CSO control projects, such as the South Boston CSO storage tunnel and the gray project adopted by the Southeast Michigan Council of Governments, which can reduce the annual CSO by 79–85% by building intercepting sewer pipes and storage facilities such as the storage tunnel [32,33]. In these projects, the construction of pipelines and storage tunnels was massive, and the storage facilities had good effects in terms of CSO control; thus, the CSO reduction rates were very high. In this study, the retrofitting of intercepting sewer pipes is one segment of the systematic CSO control scheme. Therefore, compared with the above CSO-controlled pipeline network project, the CSO reduction rate of the pipeline network in this paper is lower.

However, in the current study, there are few studies on the influence of the combination of main- and branch-intercepting sewer pipes on the control of CSO, but it has practical significance regarding engineering construction. Therefore, this paper studies the regularity of the CSO control effect of different intercepting sewer pipe construction schemes. The CSO control effects were studied by comparing schemes 1, 2, and 3 when the main-intercepting sewer pipes were unchanged and the branch-intercepting sewer pipes were increased by 100 mm. The results showed that the CSO volume decreased by 2.3% and 5.6%, respectively, when the diameter of the branch-intercepting sewer pipes increased by 100 mm. By comparing the control effects of schemes 1, 4, and 8, the regularity of the CSO control effect was studied when the branch-intercepting sewer pipes were unchanged and the main-intercepting sewer pipes were increased by 100 mm. The results showed that CSO decreased by 1.7% and 3.8%, respectively, when the diameter of the main-intercepting sewer pipes increased by 100 mm. Therefore, under the same conditions as those of scheme 1, the CSO control effect of enlarging branch-intercepting sewer pipes by 100mm was at least 1% higher than that of enlarging the main-intercepting sewer pipes by 100 mm, and the CSO control effect was weakened with the increase in the intercepting sewer pipe diameter. To verify the accuracy of the above conclusions, schemes 4, 5, 6, 7 and schemes with the same source strategies were selected to explore the above rules, and the conclusions were consistent.

The following conclusions can be drawn from the above analysis: When enlarging the intercepting sewer pipes by the same diameter, the effect on CSO control is better when enlarging branch-intercepting sewer pipes than when enlarging the main-intercepting sewer pipes. The main function of the main-intercepting sewer pipes is to transfer the sewage, and the branch-intercepting sewer pipes determine how much water is intercepted into the main-intercepting sewer pipes. As the diameter of the branch-intercepting sewer pipes increase, more sewage will be intercepted. It should be noted that enlarging the main-intercepting sewer pipes will increase the upper limit of the transfer capacity, which can improve the drainage capacity of the intercepting sewer pipe network during extreme rainfall events.

#### 4.4.2. Relationship Among Storage Tank Volume, CSO Frequency, and CSO Volume

**Figure 5:**
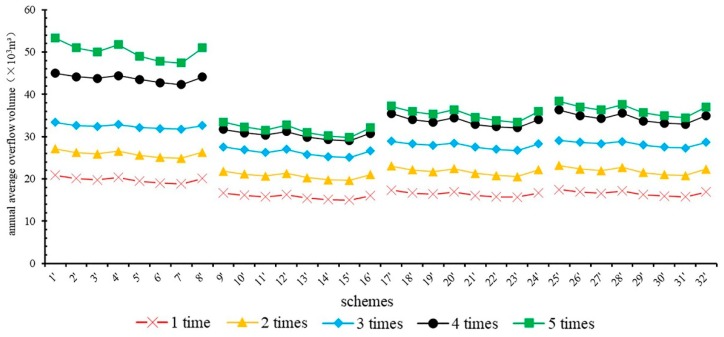
Annual average overflow under different control targets.

After adding the storage tanks, all the control schemes could achieve the control target of the annual average frequency of overflow, and different control strategies and targets affected the capacity and control effect of the storage tank. The changes in the total volume of annual overflow under the different control targets of annual overflow frequency were explored. The simulation results are shown in Figure 5.

Figure 5 shows that when the CSO control target was reduced from five times per year to four times per year, from four times per year to three times per year, from three times per year to two times per year, and from two times per year to one time per year, the average reduction in CSO volume was 2.8 × 10^3^ m^3^, 6.8 × 10^3^ m^3^, 6.1 × 10^3^ m^3^, and 5.4 × 10^3^ m^3^, respectively, with corresponding reduction rates of 7%, 19%, 21%, and 24%, respectively. This result indicates that with the improvement of the CSO control target, the CSO volume that needs to be reduced continuously increases. When the CSO control target is upgraded from four times per year to three times per year, the increase in the CSO volume that must be controlled is obviously accelerated. The above conclusions show that it is reasonable to determine the control target because the CSO frequency does not exceed four times per year.

**Figure 6:**
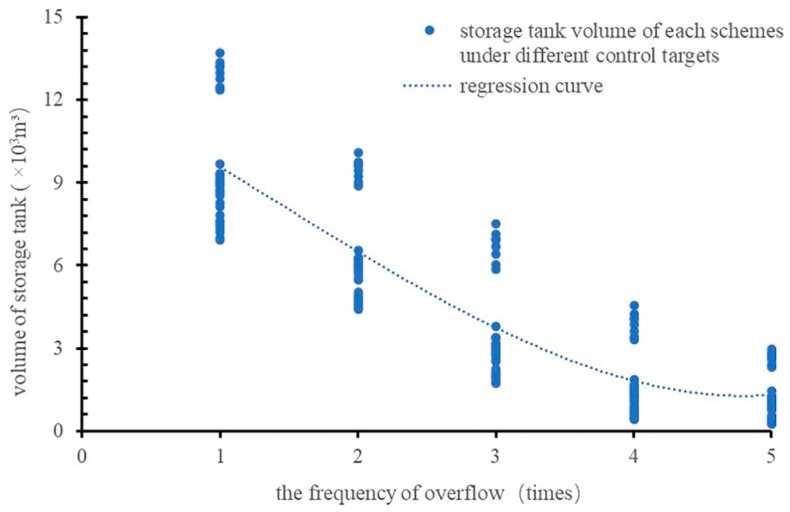
Change regulation of storage tank volumes.

In Figure 5, when the control target does not exceed five CSO events per year, the C_V_ of the curve is 0.190, and when the control target does not exceed one CSO event per year, the C_V_ is 0.098. When the CSO control targets become stricter, the increase in volume of the storage tank is different for the different schemes, which causes the differences in the annual CSO volumes between schemes to gradually be reduced. To study the change trend of storage tank volume under different control targets, the storage tank volumes under different control targets were analyzed statistically. The results are shown in Figure 6.

As shown in Figure 6, when the control target is no more than one overflow event per year, the maximum storage tank volume of the 32 schemes is 13.7 × 10^3^ m^3^. When the target of annual overflow frequency is increased to five times per year, the difference in the storage tank volumes among the 32 schemes is only 2.7 × 10^3^ m^3^. In fact, the storage capacity of LID facilities and intercepting sewer pipes is certain when different CSO control targets are adopted. When the CSO control target is upgraded from five times per year to one time per year, this approach mainly relies on increasing the volume of the storage tanks. Therefore, when the control target is strict (e.g., the number of annual CSO events is low), extreme rainfall events must be controlled, which will lead to a significant increase in the volume of the storage tank, and the storage capacity provided by the storage tank accounts for a higher proportion of the total storage capacity. With the increase in the control target of the number of annual CSO events, the volume of the storage tank is reduced, and the proportion of the storage capacity of LID facilities and intercepting sewer pipes to the total control volume gradually increases.

When the CSO control target is reduced from five times per year to four times per year, from four times per year to three times per year, from three times per year to two times per year, and from two times per year to one time per year, the increase in storage volume required to meet each reduction is 0.6 × 10^3^ m^3^, 1.8 × 10^3^ m^3^, 2.8 × 10^3^ m^3^, and 3.0 × 10^3^ m^3^, respectively, with corresponding growth rates of 42%, 99%, 77%, and 66%, respectively. Therefore, the cost of increasing the storage tank volume gradually increases. At this time, the storage tank volume increases most, and the economic benefit of improving the control target obviously decreases. The above analysis also illustrates the basis for setting the CSO control target in this study.

Figure 5 and Figure 6 show that when the control target changes, the ratio of the reduction of CSO volume to the increase in storage tank volume is the control efficiency of the storage tank. When the CSO control target is upgraded from five times per year to four times per year, the annual average CSO overflow reduction volume is 2.8 × 10^3^ m^3^, while the storage tank volume will increase by 0.6 × 10^3^ m^3^. The ratio between these two values is defined as the control efficiency of the storage tank. Therefore, the control efficiency is 5.11 when CSO control target is upgraded from five times per year to four times per year. By analogy, when the CSO control target is upgraded from four times per year to three times per year, three times per year to two times per year, and two times per year to one time per year, the control efficiency of the storage tank is 3.71, 2.17, and 1.80, respectively. This result indicates that as the CSO control target become stricter, the control efficiency of the storage tank becomes lower. Relevant research by the United States Environmental Protection Agency (USEPA) also shows that although the cost of the CSO storage tank decreases with increasing volume, it is not cost-effective in terms efficiently reducing CSO [15].

## 5. Conclusions

CSO pollution poses a serious threat to the urban water environment and is more severe in old urban areas. In order to study the CSO control strategies in the old urban area, S3 zone, a typical combined sewer area in the sponge city pilot area of Tongzhou District, Beijing, was selected as the study area. The SWMM model was used to establish the hydrologic and hydraulic model of the study area. The control target of CSO in this study was no more than four times per year. Four rainfall events were selected for calibration and verification. The calibration and validation results showed that the E_NS_ values were 0.81, 0.98, 0.74, and 0.76, respectively, which indicates that the model can be used for subsequent research and analysis.

The CSO control strategies included constructing LID facilities at the source point, retrofitting intercepting sewer pipes at the midway point, and constructing storage tanks at the end point. Four different LID construction strategies were combined with eight different pipeline network transformation strategies, and 32 sets of CSO control schemes for LID facility construction and retrofitting of intercepting sewer pipes were initially designed and simulated. Then, a storage tank was added to each scheme to achieve the control target, and the final scheme was given. This study proposes four types of strategies, which can be summarized as the "gray strategy" and the "gray-green strategies" according to the facility types of the schemes. The regularity of the CSO reduction rate in each segment of the systematic scheme was studied, and the conclusions can be drawn as follows:

(1) The LID facility has an average annual reduction rate of 22% for CSO frequency and 35% to 49% for CSO volume. LID facilities have a great control effect on the CSO volumes. Increasing the storage capacity at the source can obviously improve the CSO control effect, but its efficiency gradually decreases with increasing storage capacity, especially under the constraints of construction space. Due to the limited space available for LID facilities, the design of the LID facility’s land should be selected according to local conditions.

(2) The retrofitting of intercepting sewer pipes had an average annual reduction rate of 11% for CSO frequency and 4% to 15% for CSO volume. Increasing the interceptor capacity of the intercepting sewer pipes can improve the CSO control effect, but its control effect is lower than that of using LID facilities alone. The results showed that when enlarging the intercepting sewer pipes by the same diameter, the effect of CSO control was better when enlarging the branch-intercepting sewer pipes than when enlarging the main-intercepting sewer pipes. Therefore, in old urban areas where reconstruction is difficult, the efficiency of CSO control can be enhanced by properly enlarging the diameter of the branch-intercepting sewer pipes.

(3) The storage tanks had average annual reduction rates of 3% to 36% for CSO volume, and the reduction rate decreased with the increase in CSO volume reduction rate caused by LID facilities. Storage tank design was the last step to determine the CSO systematic control scheme in this paper, and its scale was related to the CSO control target. When the CSO control target is upgraded from five times per year to four times per year, four times per year to three times per year, three times per year to two times per year and two times per year to one time per year, the control efficiency of the storage tank is 5.11, 3.71, 2.17, and 1.80, respectively. This result indicates that a stricter CSO control target results in a lower control efficiency of the storage tank. Meanwhile, when the CSO control target was upgraded from four times per year to three times per year, both the CSO volume that needs to be controlled and the volume of the storage tank that needs to be increased are very large, which makes the cost of upgrading the control target too high, thus verifying the rationality of goal setting described in this paper. The control target must be achieved by combining the construction of LID facilities, the retrofitting of intercepting sewer pipes, and the construction of storage tanks.

When choosing a systematic control scheme for CSO in old urban areas, it is necessary to comprehensively consider the construction conditions of the old urban area, the CSO control targets, and the regularity of CSO control in each segment of the systematic scheme; then, it is necessary to design a reasonable combined scheme that considers the "source", "midway", and "end" to formulate the most suitable CSO control strategy.

It is noteworthy that the CSO control strategy can be optimized by combining model simulations with advanced algorithms and scenario analyses. Due to the published water-related permitting requirements by Tongzhou government, only the annual overflow frequency was considered as the control parameter in this study. However, when designing systematic CSO control schemes in other cities, further modifications based on this paper are needed to include additional algorithms for more complicated site situations and/or multiple design goals. In addition, simpler algorithms may exist, which will be studied in the near future, for faster and more efficient approaches. 

## Figures and Tables

**Table 1:** Rainfall event information selected by model parameter calibration.

Date of Rainfall Event	Rainfall Depth (mm)	Duration (h)	Maximum Intensity (mm/min)	Maximum Water Level (m)
20180626	20	3.5	0.20	0.81
20180707	26	16.9	0.20	0.85
20180711	30	23.8	0.11	0.52
20180716	11	3.5	0.10	0.51

**Table 2:** Determination of low impact development (LID) construction strategy.

Design Strategy	Sequence Number	Design Basis
strategy based on the goal	①	Set the control target according to the “Code for design of stormwater management and harvest engineering (DB11/685—2013)”
	②	Set the control target according to the target of 75% volume capture ratio of annual rainfall set by Tongzhou Sponge City
strategy based on the actual survey	③	Set the control target according to actual situation

**Table 3:** Strategy for retrofitting intercepting sewer pipes.

	Branch-Intercepting Pipe Diameter (mm)	500	600	700	800
Main-Intercepting Pipe Diameter (mm)					
800		5-8	6-8	7-8	-
900		5-9	6-9	7-9	8-9
1000		5-10	-	-	-

5–8 indicates the combination of the branch-intercepting pipe has diameter of 500 mm and the main-intercepting pipe has diameter of 800 mm.

**Table 4:** Combined sewer overflow (CSO) control schemes.

Type	Scheme	Source Control Strategy	Mid-way Control Strategy
Type I	1	--	5–8
	2	--	6–8
	3	--	7–8
	4	--	5–9
	5	--	6–9
	6	--	7–9
	7	--	8–9
	8	--	5–10
Type II	9	①	5–8
	10	①	6–8
	11	①	7–8
	12	①	5–9
	13	①	6–9
	14	①	7–9
	15	①	8–9
	16	①	5–10
Type III	17	②	5–8
	18	②	6–8
	19	②	7–8
	20	②	5–9
	21	②	6–9
	22	②	7–9
	23	②	8–9
	24	②	5–10
Type IV	25	③	5–8
	26	③	6–8
	27	③	7–8
	28	③	5–9
	29	③	6–9
	30	③	7–9
	31	③	8–9
	32	③	5–10

**Table 5:** Volume of storage tank.

Type I		Type II		Type III		Type IV	
Scheme	Storage tank capacity (m^3^)	Scheme	Storage tank capacity (m^3^)	Scheme	Storage tank capacity (m^3^)	Scheme	Storage tank capacity (m^3^)
1’	4600	9’	950	17’	1600	25’	1900
2’	4100	10’	750	18’	1450	26’	1700
3’	3900	11’	650	19’	1350	27’	1600
4’	4250	12’	800	20’	1500	28’	1750
5’	3650	13’	600	21’	1250	29’	1500
6’	3450	14’	500	22’	1100	30’	1350
7’	3350	15’	450	23’	1050	31’	1250
8’	4100	16’	750	24’	1450	32’	1700

’ It indicates the scheme after adding storage tank to the potential scheme.
